# A new method for *in situ* structural investigations of nano-sized amorphous and crystalline materials using mixed-flow reactors

**DOI:** 10.1107/S2053273319008623

**Published:** 2019-08-23

**Authors:** Alexandria Hoeher, Sebastian Mergelsberg, Olaf J. Borkiewicz, Patricia M. Dove, F. Marc Michel

**Affiliations:** a Geosciences, Virginia Tech, 4044 Derring Hall, 1405 Perry Street, Blacksburg, VA 24060, USA; b X-ray Science Division, Argonne National Laboratory, 9700 South Cass Avenue, Lemont, IL 60439, USA

**Keywords:** *in situ* X-ray total scattering, crystallization, amorphous calcium phosphate, amorphous calcium carbonate, pair distribution function analysis

## Abstract

Structural investigations of amorphous and nanocrystalline phases forming in solution are historically challenging. Few methods are capable of *in situ* atomic structural analysis and rigorous control of the system. A mixed-flow reactor (MFR) is used for total X-ray scattering experiments to examine the short- and long-range structure of phases *in situ* with pair distribution function (PDF) analysis. The adaptable experimental setup enables data collection for a range of different system chemistries, initial supersaturations and residence times. The age of the sample during analysis is controlled by adjusting the flow rate. Faster rates allow for younger samples to be examined, but if flow is too fast not enough data are acquired to average out excess signal noise. Slower flow rates form older samples, but at very slow speeds particles settle and block flow, clogging the system. Proper background collection and subtraction is critical for data optimization. Overall, this MFR method is an ideal scheme for analyzing the *in situ* structures of phases that form during crystal growth in solution. As a proof of concept, high-resolution total X-ray scattering data of amorphous and crystalline calcium phosphates and amorphous calcium carbonate were collected for PDF analysis.

## Abstract 2

A novel method is introduced for *in situ* X-ray total scattering experiments. Two examples of the method as applied to non-classical nucleation and crystal growth studies are discussed.

## 1. Introduction   

Investigations of mineral growth in solution have identified a new paradigm of crystallization that occurs through reactive intermediates (Niederberger & Cölfen, 2006 ▸; Gebauer & Cölfen, 2011 ▸; De Yoreo *et al.*, 2015 ▸). Of particular interest are the amorphous materials and nanoparticles that can dissolve and reprecipitate or aggregate to form crystalline structures. Recent attempts to establish the short-range structure and evolution pathways of these phases have faced challenges due to the metastable nature of these phases (Goodwin *et al.*, 2010 ▸; Reeder & Michel, 2013 ▸; Schmidt *et al.*, 2014 ▸; Posner & Betts, 1975 ▸; Yin & Stott, 2003 ▸; Du *et al.*, 2013 ▸; Habraken *et al.*, 2013 ▸; Reeder *et al.*, 2013 ▸). Initial efforts physically stabilized samples, for example by isolating the sample from solution by freeze-drying (Posner & Betts, 1975 ▸; Meyer & Eanes, 1978 ▸). This approach inhibits the crystallization process long enough for analysis and enables investigative techniques that are not compatible with solutions. Drying a sample, however, can cause structural alterations (Eichert *et al.*, 2003 ▸), and does not easily allow for further investigations of sample evolution over time. Additionally, sublimation of the water can cause counter-ions to precipitate additional phases that interfere with the analysis of the metastable intermediate. In response to these limitations, there are an increasing number of *in situ* structural investigations of amorphous phases, primarily by examining aliquots (Du *et al.*, 2013 ▸; Zhang *et al.*, 2015 ▸; Borkiewicz *et al.*, 2010 ▸). These often small-scale experiments lack rigorous control of the solution conditions and there is the potential for samples to structurally evolve during data collection. To address these challenges, we adapted the mixed-flow reactor (MFR) design documented by Blue *et al.* (2013 ▸) to conduct structural investigations of amorphous and crystalline mater­ials. The MFR approach allows us to control solution chemistries and supersaturations, precipitate samples under steady-state conditions, create highly reproducible samples, and maintain precise control of the sample age during data collection (Blue *et al.*, 2017 ▸).

Here we present a powerful method for *in situ* structural analysis that is easily adapted to various systems. Using the calcium phosphate and calcium carbonate systems as a proof of concept, we focus on collecting synchrotron total X-ray scattering data for pair distribution function (PDF) analysis. This includes both Bragg and elastic diffuse scattering, allowing us to examine the structure of materials that only have short-range order (*e.g.* nano-sized and/or amorphous substances) and materials with long-range order (*e.g.* micro- and macrocrystalline minerals) using the same analytical technique. Suspensions of crystalline and amorphous mater­ials that are synthesized in the MFR proceed directly through the X-ray beam in order to allow us to collect and compare structures of the samples. We present the steps performed to optimize data extraction for high-quality *in situ* structural data of both well- and poorly ordered materials. Examples of data collected in the calcium phosphate and calcium carbonate systems are included. Our approach also offers the potential to examine a series of different residence times and capture structural transformations that occur throughout the crystallization process. This adaptable procedure provides a new approach for collecting high-quality, reproducible structural information from *in situ* crystallization experiments.

## 2. Mixed-flow reactors for total X-ray scattering   

**Figure 1:**
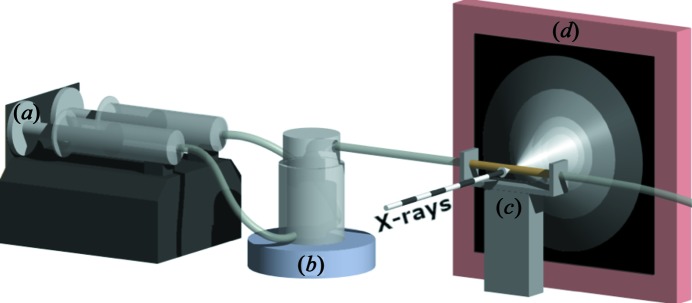
A schematic of the experimental setup used at Sector 11-ID-B of the APS. (*a*) Syringe pump with two syringes, (*b*) mixed-flow reactor on a stir plate, (*c*) X-ray scattering window and (*d*) 2D detector.

The basic design of the MFR method is presented in the work of Blue *et al.* (2013 ▸, 2017 ▸). A high-precision syringe pump (Harvard Apparatus PHD ULTRA 4400) is used to continually pump the reactant solutions (Sigma–Aldrich ACS reagent CaCl_2_ and Na_2_HPO_4_ in 18 MΩ ultra-pure water) into the MFR. A stir bar placed within a well at the base of the reactor provides steady mixing (800 r min^−1^). As mixing proceeds, the solution within the reactor becomes supersaturated and precipitation occurs continuously for the duration of mixing. As the MFR internal volume is filled, the well-mixed suspension flows out of a single opening at the top and through the scattering window, a Kapton capillary (3 mm diameter with 0.05 mm wall thickness), which intersects the path of the X-ray beam (Fig. 1 ▸).

**Figure 2:**
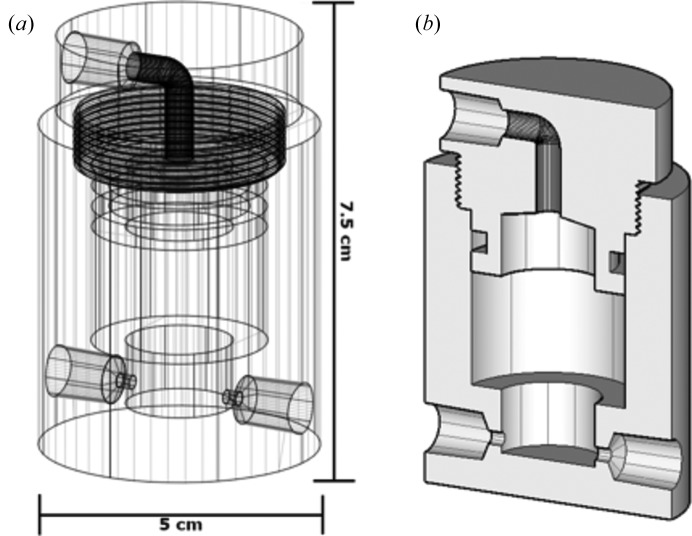
Schematics of the mixed-flow reactor shown as (*a*) a wire frame of the entire reactor and (*b*) a rendered cross section. Solutions flow into the reactor through the two inlets at the bottom (tubing connections not shown). A small stir bar in the well at the bottom of the reactor mixes the solutions as they enter the reactor and they exit through the outlet at the top. The reactor has an internal volume of about 25 ml.

The experiment design is the same for all of the samples collected. The pump creates a precise flow rate of 3 ml min^−1^ for a 5 min residence time (see discussion below). The Monoject 140 cm^3^ piston syringes are modified with custom Teflon plunger caps and connect to the reactor with fluorin­ated ethylene propylene (FEP)-lined Tygon SE-200 tubing [Cole-Palmer, 1/8′′ (3.2 mm) inner diameter] via two ports at the base of the reactor. Additional tubing connects the reactor output to the Kapton scattering window, where X-ray scatter from the capillary is collected by the detector. The effluent then proceeds to a waste container. The reactors are manufactured from a cast acrylic rod and contain an internal volume of ∼25 ml (Fig. 2 ▸). Similar reactors with modified designs have been produced by desktop stereolithography 3D printing, providing a rapid and cost-effective alternative to creating the reactors via traditional machining (Michel *et al.*, 2018 ▸).

The age of the sample when it passes through the beam is controlled by the residence time, the average time from initial mixing, until the mixed solution suspension flows into the X-ray scattering window. Hydraulic residence time (τ) is calculated,
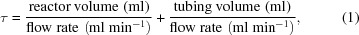
and is adjusted by changing the flow rate. As long as the solution is still flowing, and the sample remains suspended, the residence time and the age of the sample passing through the X-ray beam remain constant. Once the solution exits the reactor and flows through the tubing and scattering window, it behaves as a plug-flow reactor. Minimizing the tubing length helps to control the residence time and reduce the duration of time spent in the plug-flow system.

## 3. Data acquisition   

### 3.1. Beamline specifications   

Collection of total X-ray scattering data for PDF analysis took place at Sector 11 of the Advanced Photon Source (APS), Argonne National Laboratory. In-depth explanations of the PDF technique may be found elsewhere (Proffen *et al.*, 2003 ▸, 2005 ▸; Reeder & Michel, 2013 ▸; Egami & Billinge, 2003 ▸). In its current configuration, 11-ID operates three individual end-stations simultaneously for 100% of the time. X-rays are delivered through a combination of two in-line undulators. One, a 3.3 cm device, delivers X-rays to the main branch and end-station 11-ID-D. The second, a 2.3 cm device, is used for the side branch, plus two end-stations: 11-ID-B and 11-ID-C. A double-crystal Si (111) vertical-bounce monochromator is used for the main branch to deliver X-rays in the energy range of 6–30 keV. Two individual, in-line, bent-Laue brilliance-preserving monochromators are used to distribute X-rays to 11-ID-B and 11-ID-C. A symmetrically cut Si crystal is used to generate energies of 58.6 keV (Si 311, fifth harmonic of the undulator) and 85.6 keV (Si 422) at 11-ID-B and an asymmetrically cut Si crystal is used at 11-ID-C to deliver X-rays of 105.6 keV (Si 311, ninth harmonic).

All experiments were performed at 11-ID-B. At 11-ID-B, an amorphous silicon-based area detector [2048 × 2048 pixel Perkin-Elmer (Chupas *et al.*, 2007 ▸)] was positioned about 16 cm from the samples to collect total scattering intensity data. A cerium dioxide standard (CeO_2_, NIST diffraction intensity standard set 674a) mixed with glassy carbon (ratio ∼1:25 CeO_2_ to carbon) was used to calibrate the sample–detector distance, beam energy, and beam center and non-orthogonality. Data were primarily collected during top-up mode and results were highly reproducible using this technique, regardless of mode (top-up or non-top-up).

### 3.2. Data optimization   

A crucial part of data collection involves minimizing noise from the detector. The signals for *in situ* samples, especially for syntheses involving low-*Z* elements, such as carbon, oxygen, calcium and phospho­rous, are quite weak and consist mostly of parasitic background scattering. The detector poses the highest potential risk of error in data collection for these experiments. A common problem is the overexcitation of pixels, which results in a false positive signal from the detector and can affect sequential data scans (Skinner *et al.*, 2012 ▸).

To minimize overexcitation, the exposure times are kept short, minimizing detector saturation. A single scan is an average of 200 frames, each frame based on 0.3 s of beam exposure, which amounts to 1 min total, per scan. Every five scans, we collect a dark current scan, in which the beam is turned off for 1 min. This allows the detector to rest, removes residual intensity and prevents overexcitation. The dark currents are subtracted from the raw patterns automatically within the *QXRD* program (Jennings, 2010 ▸). Intense scattering signals that occur for crystalline materials with high electron density are most likely to produce overexcited pixels in the detector. The duration of beam exposure is shorter for well-crystalline samples than for amorphous or poorly crystalline samples. To reduce the chance that overexcited pixels will create false intensities in sequential experiments, total scattering data are first collected for all amorphous, then poorly crystalline, then well-crystalline samples.

**Figure 3:**
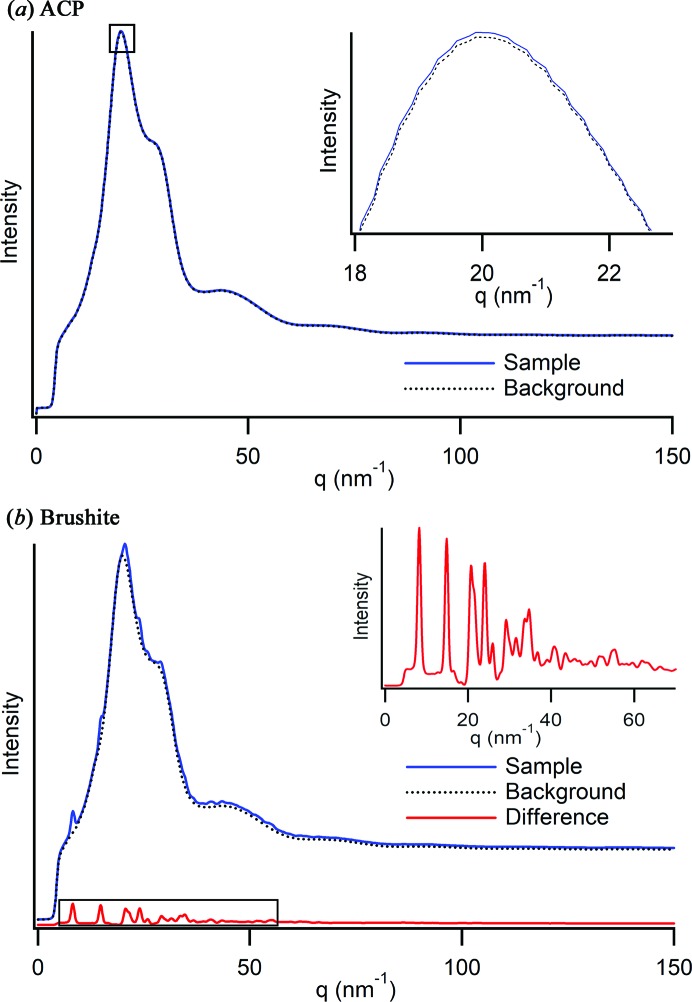
Integrated scattering data and the water background that is subtracted from the data presented for (*a*) an amorphous calcium phosphate (ACP) profile with 97% of the total scattering signal attributed to background signal. An enlargement of the profile at its maximum intensity (inset) shows the minimal difference between the sample and background signal. In (*b*), 97.9% of the total intensity of a brushite profile is background. The red profile (inset) is the difference between the two profiles, which produces indexable peaks.

After data collection, the *FIT2D* program (Hammersley *et al.*, 1996 ▸) is used to average scans and convert the data from 2D TIF images to 1D CHI files. The masks used to remove dead pixels and the beamstop from data integration and the effects of different masks on signal noise are outlined in Section S1 of the supporting information. *xPDFsuite* (Yang *et al.*, 2014 ▸; Juhás *et al.*, 2013 ▸) is then used to subtract the background for the samples and to perform Fourier transformation of the total scattering data for PDF analysis. Typical *Q*_max_ values achieved in our experiments are between 18 and 22 Å^−1^. To optimize the quality of the data collected using the MFR, background subtraction is the most critical stage of data processing. For this type of system, the background-to-sample ratio is substantially higher than for traditional *ex situ* analysis methods. The contribution of amorphous or crystalline precipitate is often only between 1 and 3% of the total X-ray scattering signal (Fig. 3 ▸). A background subtraction with precision in the third or fourth decimal place is commonly needed to eliminate under- or over-subtraction errors for amorphous samples.

**Figure 4:**
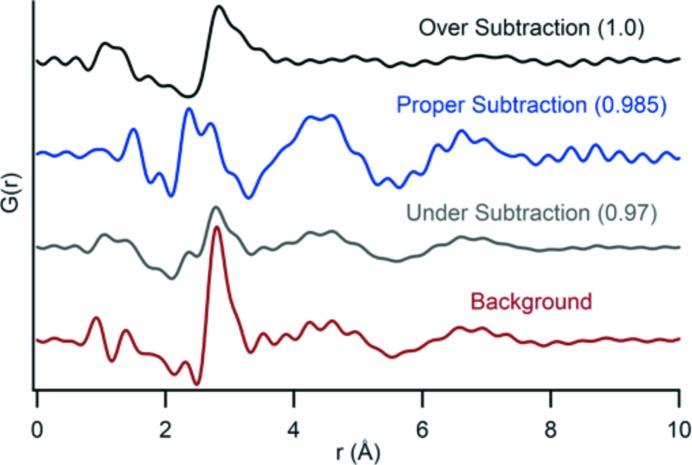
A series of PDF profiles show the consequences of over- or under-subtraction of an ACP data set. In this example, subtraction that is off by only ±0.015 results in a profile more similar to the water background and eliminates some of the major structural features.

Over- or under-subtraction can result in a PDF profile consistent with that of water, instead of the signal from the sample (Fig. 4 ▸). Because the necessary precision in background subtraction is so high, each data set needs to be closely evaluated and adjusted individually. A universal background subtraction value for a suite of samples under our conditions is not possible. It could be considered under different conditions where a sample produces a higher signal-to-background ratio. Under ideal conditions, *i.e.* using top-up mode and identical exposure times for the sample and blank, the multiplication factor between the sample and background equals one. In most cases, however, there is deviation from this value due to minor variations in beam intensity, even during top-up mode. If top-up mode is not available, normalizing the sample and background data at high *Q* values helps to determine an appropriate scaling and background subtraction.

**Figure 5:**
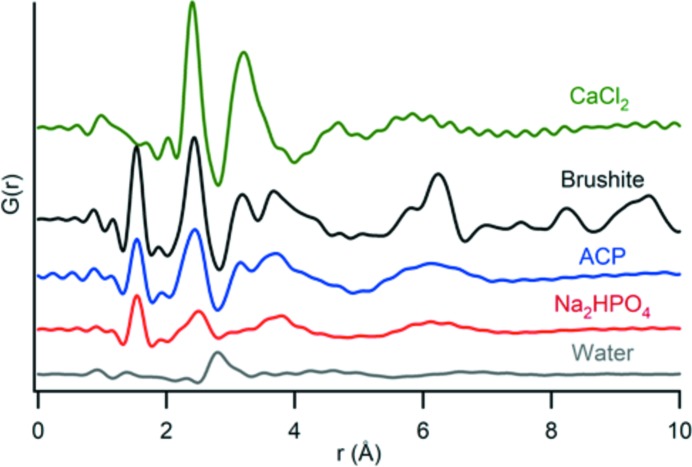
Comparison of the PDF data collected for ACP and brushite, and different considered backgrounds: CaCl_2_, Na_2_HPO_4_ and deionized water. The first two peaks in the ACP and brushite data represent mainly the P–O and Ca–O distances, with some O–O contributions. The PDF profiles for CaCl_2_·H_2_O and Na_2_HPO_4_ have similar locations for these initial peaks, but ACP and brushite also contain structure that is not inherited from the reactant solutions. The water PDF does not have a peak in the same location as peaks in ACP and brushite, ensuring that no signal from the sample is accidentally subtracted.

Choosing an appropriate background for subtraction is critical for data optimization with *in situ* experiments. Different potential backgrounds are considered, including a capillary of the same size filled with deionized water and a capillary filled with either of the reactant solutions (Fig. 5 ▸). Because solution concentrations of ions from the low-solubility calcium phosphate and calcium carbonate phases are below 50 m*M*, we find the signal from deionized water most suitable for background subtraction. If this technique is used on samples far from steady state or samples with higher solubility, using a reactant solution as a background signal may be necessary. For each sample, background and *in situ* signals are collected using the same number of scans. The comparison of PDF profiles of reagent solution with those of the precipitate samples is also an important proof of concept to evaluate data quality and to check for inherited structural elements (Fig. 5 ▸).

It should be noted that all background subtractions are performed in reciprocal space because the focus of this study is the total structure of our samples and all atom pairs. It is possible to perform background subtractions in real space; however, this is typically done for studies of differential PDFs focusing on a specific chemical species. Examples of this are antimonate and arsenate speciation, as outlined by van Genuchten & Peña (2016 ▸) and adsorbed species on ferri­hydrite by Harrington *et al.* (2010 ▸) and Zhu *et al.* (2014 ▸). Detailed methods and considerations for this type of processing can be found in the work of Wurden *et al.* (2010 ▸).

The user input compositions within *xPDFsuite* have little impact on the final structure compared with other data processing software, such as *PDFgetX2* (Qiu *et al.*, 2004 ▸). This is advantageous for materials without a well-defined chemical composition, such as the amorphous intermediates in this study. Despite this input flexibility, assigning a composition with elements or compounds not found in your sample produces a PDF profile with features that are not real. For amorphous calcium phosphate (ACP) materials, a composition of Ca_9_(PO_4_)_6_ is used. This is a widely applied composition for calcium phosphate materials with the short-range order seen in ACP (Posner & Betts, 1975 ▸; Betts *et al.*, 1975 ▸). For crystalline phases their theoretical chemical compositions are used, which is CaHPO_4_·2H_2_O for brushite. The limited variables available in *xPDFsuite* make data extraction straightforward once a suitable background subtraction value is achieved.

### 3.3. Special considerations for MFR samples   

**Figure 6:**
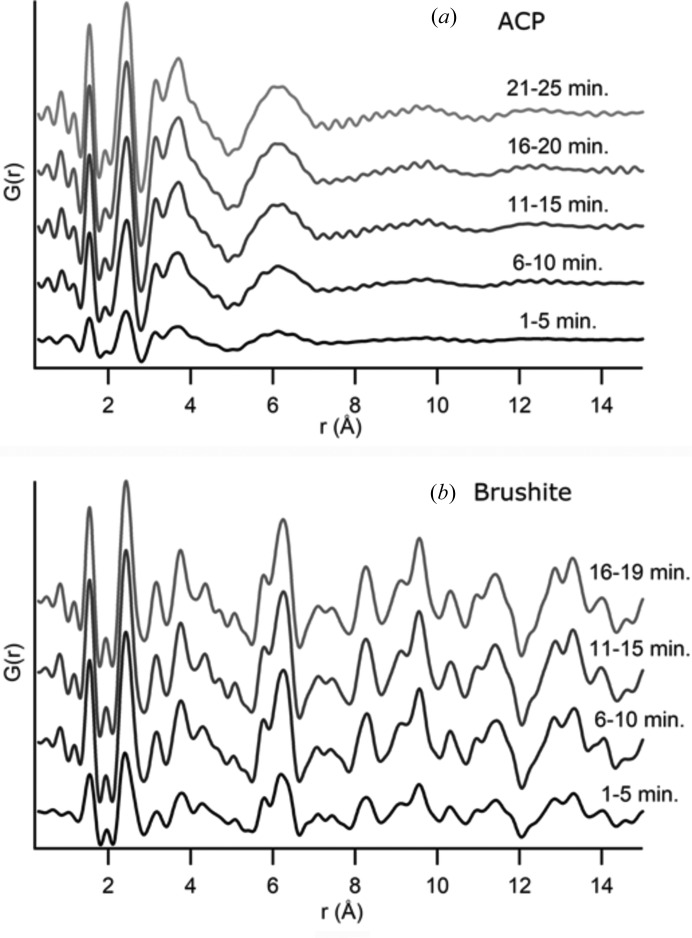
To demonstrate that samples do not evolve during data collection, the PDF data for a sample of (*a*) ACP and (*b*) brushite are shown as sequential averages of five scans. The structure remains consistent across the scans, but there is an increase in overall intensity for both samples after the first five scans. During these first five scans (the first 5 min of data collection), the solution inside the MFR is still approaching steady state and there is a lower particle density leading to a less intense scattering signal.

Further optimization of PDF profiles can be performed by carefully evaluating the timescales needed for each experiment and adapting X-ray exposures accordingly. For example, simple mixing theory shows that it takes approximately three residence times for the MFR system to reach steady state (Jensen, 2001 ▸). This can vary for different experiments depending upon the concentration of particles produced and the density of the solids (Jensen, 2001 ▸). Fig. 6 ▸ shows PDF data for ACP [Fig. 6 ▸(*a*)] and brushite [Fig. 6 ▸(*b*)] that were both synthesized with τ = 5 min. The data are presented as a series of 5 min averages. The major peak locations are visible in all the profiles, but data from the first 5 min of data collection show a much lower intensity. Sequential groups of averaged time-points present no significant differences, which means we achieve steady state between one and two residence times. It also provides evidence that the structure is not evolving during data collection and no features are being averaged out. All subsequent data were collected with a minimum data collection time equivalent to 2τ (at least 10 min for data shown here, but variable depending on flow rate).

**Figure 7:**
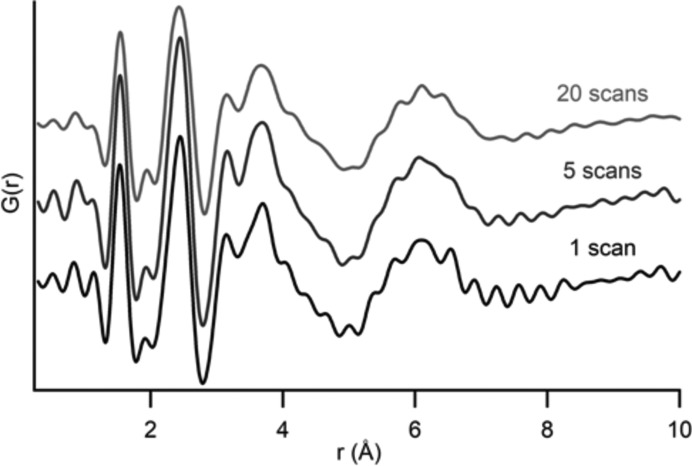
Averaging multiple scans of data contributes to PDF profiles that have a reduced amount of noise. A single sample of ACP is presented as a single scan, an average of five and an average of 20 scans. The noise in the data is significantly reduced between one and five scans. There is a slight reduction in noise between five and 20 scans. Averages beyond 20 scans did not have a significant reduction in noise. Each scan represents 1 min of data collection, indicating noise reduction is optimized with at least 20 min of collection time.

Materials with low scattering intensity require further data optimization by averaging multiple scans from a single sample to minimize Fourier noise. Fig. 7 ▸ shows a significant reduction in noise between a single scan and an average of five scans for the ACP example. Further noise reduction by averaging 20 scans is not significant when compared with the five scan averages. Use of more than 20 scans to produce one average does not significantly improve the overall quality of the data.

Note that the data collection times and number of scans per sample depend upon residence time. Data are collected while the sample flows through the X-ray window at constant speed. Short residence times (faster flow rates) produce few scans, potentially too few to yield a signal of sufficient quality. There is also an upper limit to the maximum practical residence times. Low pumping speeds (τ > 25 min) cause solids to settle both in the reactor and the tubing, even at high rates of stirring (800 r min^−1^). This blocks the solution flow at the connector transition between the tubing and the scattering window. Settling also leads to variable age in the sample. The supersaturation of the system and the amount of precipitate can vary widely and have a significant impact on the upper range of residence time for an experiment. Varying experimental design by altering the initial volume of reactant, the reactor volume and tubing inner diameter expands the accessible range of residence times.

To test the limitations of the device, we performed experiments for τ = 2–25 min. We found a residence time of 5 min (∼25 scans and 5τ) was suitable for investigating the structures that dominate early stages of crystal growth. At this residence time we captured amorphous calcium carbonate (ACC) as well as ACP and brushite. The presence of ACP or brushite could be controlled by altering the initial system conditions (*e.g.* concentration and pH).

## 4. PDF analysis of *in situ* samples   

**Figure 8:**
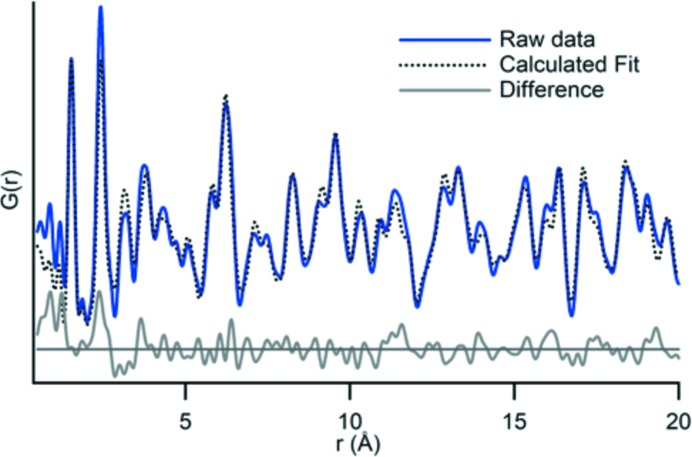
PDF data of an *in situ* brushite sample, modeled against a published brushite structure. The fit was determined by calculating a theoretical PDF from the published structure, allowing for some parameter refinement, and comparing it with the observed PDF data. The difference between the observed and the fitted data is random and mostly oscillates near zero. At low *r* (*x* axis), where the signal contains artifacts of the Fourier transform, the errors in peak positions and intensities are high.

Our adaptation of the MFR design allows us to obtain high-quality total X-ray scattering data for PDF analysis of *in situ* crystalline and amorphous phases. To identify and structurally refine the collected phases a theoretical PDF is calculated from a published structure file using *PDFgui* (Farrow *et al.*, 2007 ▸). The crystalline calcium phosphate phase was identified as brushite through comparison with a structure published by Sainz-Díaz *et al.* (2004 ▸). We refined unit-cell dimensions, the linear atomic correlation factor, phase scale factor, thermal parameters, atom positions and instrument damping (Section S2 in the supporting information). Fig. 8 ▸ compares the PDF of the collected data with the refined calculated PDF. There is a 30% residual misfit between these two data sets. While this would be considered a fairly high residual for a well-crystalline powder, we justify this residual based on the intricacies of the data extraction and large opportunities for error in background subtraction. The fit accounts for all major peaks and features in the profile. A concentrated contribution of misfit between the sample and the calculated PDF originates in the Fourier noise at the beginning of the profile, which likely does not correspond to real features. The first structural peak for the calcium phosphate system is the P–O peak at 1.56 Å. If the profiles are compared without considering any signal below 1.3 Å, the residual drops to 25%.

A previous study (Proffen *et al.*, 2005 ▸) identified concurrent amorphous and crystalline phases in the same sample by examining the difference profile after fitting the crystalline phase. It is possible that there is ACP mixed with brushite in this sample, but we do not find evidence for it in the difference profile. The misfit appears random and centered around zero. With the low signal-to-noise ratio for these samples, a degree of noise is expected and also contributes to the overall error.

**Figure 9:**
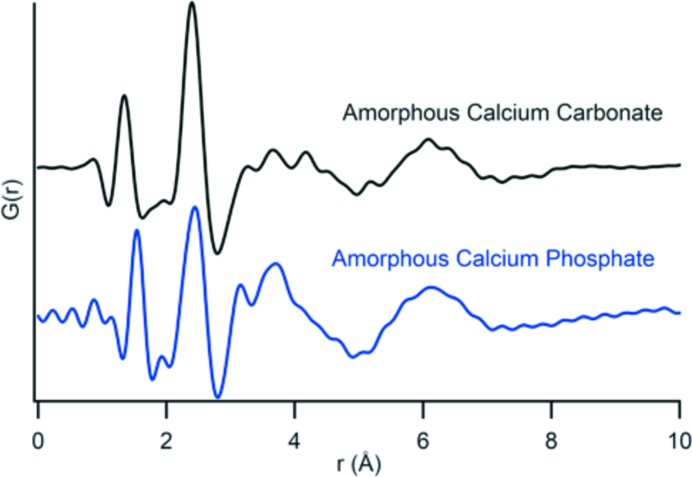
*In situ* PDF data from ACC and ACP synthesized in the MFR. There are distinct differences between the phases, most notably in the position of the first peak at 1.6 Å for the P–O distances in ACP and at 1.3 Å for the C–O distances in ACC.

The MFR is also used to collect *in situ* PDF data for ACC, demonstrating the adaptability of the MFR to address questions in various chemical systems. A separate reactor is used for all calcium carbonate experiments to avoid cross-contamination. The synthesis of ACC involves mixing CaCl_2_ and MgCl_2_ solutions with a pH-adjusted NaHCO_3_ solution in the MFR (Sigma–Aldrich ACS reagent, pH = 9.5, Mg/Ca = 1.93 in solution). The process for data collection and extraction developed for ACP can be applied to ACC samples and Fig. 9 ▸ compares the quality of PDF profiles produced. A notable difference between the two profiles is the location of the first peak, at ∼1.3 Å, due to the shorter average bond length C—O in carbonate than is seen in the average P—O length of phosphate. This integrated area of these peaks is also different, due to differences in coordination number. In contrast, the second peak for both ACC and ACP is at ∼2.4 Å because the Ca—O bond distance is the same for both phases. The profile contributions beyond the first two peaks are more convoluted, but certain structural influences can still be interpreted. Monodentate or bidentate bonding geometries, especially between Ca–P in ACP and Ca–C in ACC can affect the structure and be reflected in peak positions. The C–C distances in a structure with trigonal planar CO_3_ will differ from the P–P distances with PO_4_ tetrahedra. These predictable contributions can help further interpretation of complex profiles.

Comparing the PDF profiles of ACP and ACC in Fig. 9 ▸ with examples of ACP from Posner & Betts (1975 ▸) and ACC in the work of Michel *et al.* (2008 ▸) shows the Ca–O, P–O and C–O peak locations are consistent between our samples and the literature. Both *in situ* PDF profiles suggest a shorter range of structural coherency than is described in the *ex situ* literature. The *Q*_max_ for our ACP sample is 20 Å^−1^, while Posner & Betts (1975 ▸) terminated their data at 13 Å^−1^, resulting in significant differences between the samples. Our ACC profile is very similar to the one presented by Michel *et al.* (2008 ▸). There are some differences between 3 and 5 Å, potentially from magnesium in the synthesis and contributions from associated water.

The strength of this method is that we can examine the structures of *in situ* precipitates for a wide variety of different chemical systems simply by minor variations in the initial setup. By changing our initial solutions, we examined the structural variations between ACC and ACP. Similarly, experiments could be designed to investigate the structural effects of other influences on ACC and ACP such as pH, supersaturation and ion substitution. Combining MFRs with total X-ray scattering allows us to investigate a range of structure and evolution questions that are difficult, if not impossible, to answer using other techniques.

## 5. Conclusions   

We use an MFR-based experimental design that can be combined with X-ray total scattering techniques for *in situ* investigations of crystal growth pathways. The reactor allows precise control of system chemistry and residence times and is appropriate for investigations of various chemical systems where the reaction time of interest is between 2 and 25 min. This technique enables structural examination of samples in suspension, without the sample chemistry or structure evolving during data collection. Data are collected soon after initial mixing and sample precipitation, allowing investigations into initial phase formations and structural developments that occur in the crystallization process. Optimizing data collection and processing alongside collecting proper background data and careful background subtraction is noted as a key step.

We have collected structural information on crystalline and amorphous phases *in situ* for both the calcium carbonate and calcium phosphate systems using the MFR. An *in situ* crystalline sample was successfully modeled in real space against a published structure for brushite. The amorphous profiles showed similarities with their crystalline counterparts, which allowed us to infer short-range structural information for these phases, without performing modeling. MFRs will allow us to investigate the structural effects of different initial variables on these materials that have previously proven challenging to examine.

This new technique presents an opportunity to improve data collection on the structures of amorphous and poorly crystalline phases, leading to more robust structural models. Results from MFR experiments will need to be combined with other techniques before a comprehensive picture of the structure of these complex amorphous materials can be reached. Future efforts will also focus on expanding the range of residence times that are possible and adding adaptations such as those that will allow us to monitor pH or ion concentrations during the reaction.

## Supplementary Material

Masking details and refinement tables. DOI: 10.1107/S2053273319008623/sc5131sup1.pdf
