# Supplementary material for: A new method for in situ structural investigations of nano-sized amorphous and crystalline materials using mixed-flow reactors
Source: Acta Crystallogr A Found Adv. 2019 Aug 23;75(Pt 5):758–65. doi: 10.1107/S2053273319008623 (PMC6718202; doi:10.1107/S2053273319008623)
Supplement: Supplementary file 1 [file a-75-00758-sup1.pdf]

Acta Cryst A
FOUNDATIONS
ADVANCES

Volume 75 (2019)

Supporting information for article:

A new method for *in situ* structural investigations of nano-sized amorphous and crystalline materials using mixed-flow reactors

Alexandria Hoeher, Sebastian Mergelsberg, Olaf J. Borkiewicz, Patricia M. Dove and F. Marc Michel

S1. Masking

Masking the beam stop, major dead zones, and the edge of the detector (where the angle of the beam is very shallow) are typical practices (**Figure 1**). With the extremely low signal to noise ratio present in our amorphous samples we created a more extensive mask than normal in an attempt to reduce the amount of noise seen in the raw data at higher momentum transfer values. Typical Qmax values achieved in our experiments are between 18 – 22 $Å^{-1}$. As can be seen in **Figure 2**, the *F(q)* of amorphous samples shows significantly reduced noise when the extensive mask is applied. The decrease in noise does not have a significant effect on the resolution or observed structure of our samples after undergoing a Fourier transform to obtain the PDF (*G(r)*). In addition, for a crystalline brushite sample, there appears to be no difference in the noise, resolution, or structure of the samples before or after the Fourier transform, regardless of which mask is used. Minimizing excess noise is important for accurate data analysis, but this comparison shows that the excess noise from the unmasked dead pixels is only observable in amorphous samples and doesn't impact the final PDF profile of a sample.

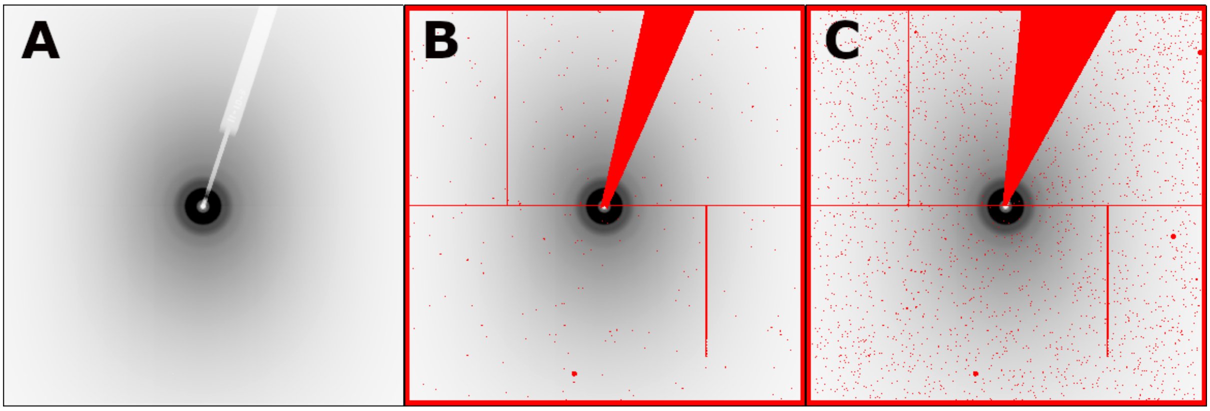

Figure S1 (A) The raw signal collected on the 2D detector includes a shadow of the beam stop which in (B) and (C) is overlain with a red mask that covers unwanted signals and prevents their inclusion in data integration. Unwanted signals include the beam stop, dead pixels or zones, and the edge of the detector (where the angle at which the beam interacts with the detector is shallow). The mask in (B) is typically used at Sector 11 ID-B and covers the most significant and obvious dead zones and pixels on the detector. The mask in (C) is more expansive, blocking out a significantly greater number of dead and anomalous pixels. Additionally, for both (B) and (C) intensity data collected in the corners of the detector are not used because the radial integration in these regions is incomplete.

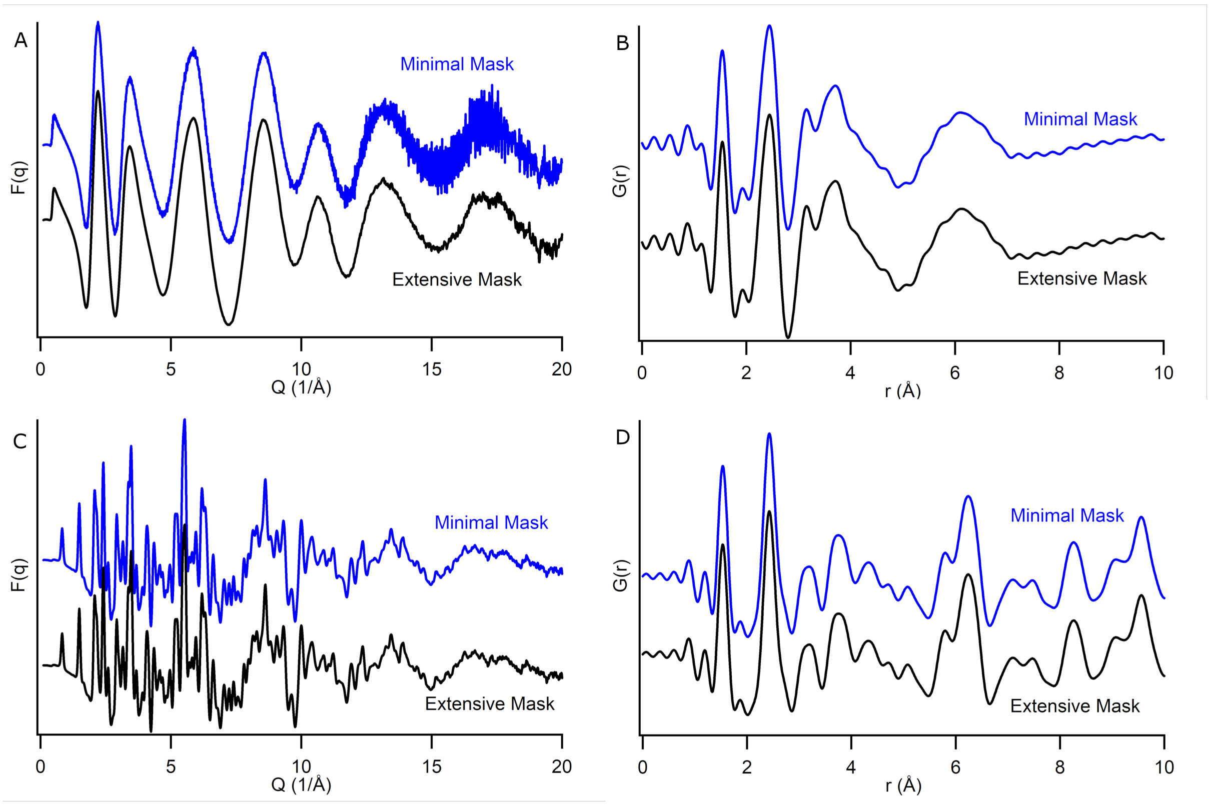

Figure S2 Using a more extensive mask with amorphous samples (A and B) greatly reduces the amount of noise seen at high Q^{-1} in the F(q) (A), but has no impact on the PDF profile after the data undergoes a Fourier transform (B). For a sample of crystalline brushite (C and D) there is no significant difference in the data processed with a minimal or extensive mask applied.

S2. Brushite Modeling Refinements

The refinement outputs for the *in situ* brushite modeling in PDFgui, presented in this paper are listed here. Atom position refinements (**Table 1**), anisotropic temperature factors (**Table 2**), unit cell parameters, scale factor, and quadratic correlation parameters (**Table 3**) are all listed below.

Table S1 A list of refined atom positions.

Atom	Position		
	X	Y	Z
Ca	0.49	0.67	0.75
Ca	0.49	0.33	0.25
Ca	0.99	0.17	0.75
Ca	0.99	0.83	0.25
P	0.01	0.68	0.77
P	0.01	0.32	0.27
P	0.51	0.18	0.77

P	0.51	0.82	0.27
O	0.76	0.64	0.53
O	0.76	0.36	0.03
O	0.26	0.14	0.53
O	0.26	0.86	0.03
O	0.91	0.72	0.93
O	0.91	0.28	0.43
O	0.41	0.22	0.93
O	0.41	0.78	0.43
O	0.20	0.59	0.84
O	0.20	0.41	0.34
O	0.70	0.09	0.84
O	0.70	0.91	0.34
O	0.12	0.74	0.65
O	0.12	0.26	0.15
O	0.62	0.24	0.65
O	0.62	0.76	0.15
O	0.27	0.57	0.40
O	0.27	0.43	0.90
O	0.77	0.07	0.40
O	0.77	0.93	0.90
O	0.69	0.55	0.01
O	0.69	0.45	0.51
O	0.19	0.05	0.01
O	0.19	0.95	0.51
H	0.71	0.67	0.37
H	0.71	0.33	0.87
H	0.21	0.17	0.37
H	0.21	0.83	0.87
H	0.25	0.51	0.39
H	0.25	0.49	0.89
H	0.75	0.01	0.39
H	0.75	0.99	0.89
H	0.25	0.59	0.21
H	0.25	0.41	0.71
H	0.75	0.09	0.21
H	0.75	0.91	0.71
H	0.72	0.49	1.00
H	0.72	0.51	0.50
H	0.22	0.99	1.00
H	0.22	0.01	0.50
H	0.89	0.57	0.15
H	0.89	0.43	0.65
H	0.39	0.07	0.15
H	0.39	0.93	0.65

Table S2 The anisotropic temperature factors used for modeling atoms in general positions

Atom	Anisotropic Temperature factor
Ca	0.006
P	0.004
O	0.015
H	0.010

Table S3 The unit cell dimensions (Å, °) and other factors used to refine the brushite model.

Scale factor	0.119
Linear correlation factor	0
Quadratic correlation factor	1.49
a	6.37
b	15.2
c	5.80
α	90
β	119
γ	90
